# Predation Risk Shapes Social Networks in Fission-Fusion Populations

**DOI:** 10.1371/journal.pone.0024280

**Published:** 2011-08-30

**Authors:** Jennifer L. Kelley, Lesley J. Morrell, Chloe Inskip, Jens Krause, Darren P. Croft

**Affiliations:** 1 School of Animal Biology, University of Western Australia, Perth, Western Australia, Australia; 2 Department of Biological Sciences, University of Hull, Hull, United Kingdom; 3 Durrell Institute of Conservation and Ecology, University of Kent, Kent, United Kingdom; 4 Leibniz-Institute of Biology and Ecology of Fishes, Berlin, Germany; 5 Institute of Integrative and Comparative Biology, University of Leeds, Leeds, United Kingdom; 6 School of Psychology, University of Exeter, Exeter, Devon, United Kingdom; University of Western Ontario, Canada

## Abstract

Predation risk is often associated with group formation in prey, but recent advances in methods for analysing the social structure of animal societies make it possible to quantify the effects of risk on the complex dynamics of spatial and temporal organisation. In this paper we use social network analysis to investigate the impact of variation in predation risk on the social structure of guppy shoals and the frequency and duration of shoal splitting (fission) and merging (fusion) events. Our analyses revealed that variation in the level of predation risk was associated with divergent social dynamics, with fish in high-risk populations displaying a greater number of associations with overall greater strength and connectedness than those from low-risk sites. Temporal patterns of organisation also differed according to predation risk, with fission events more likely to occur over two short time periods (5 minutes and 20 minutes) in low-predation fish and over longer time scales (>1.5 hours) in high-predation fish. Our findings suggest that predation risk influences the fine-scale social structure of prey populations and that the temporal aspects of organisation play a key role in defining social systems.

## Introduction

Linking individual-level behaviours with large-scale social structure is fundamental to ecology and evolution because it allows us to understand patterns of gene, information or disease transfer through populations, predict the impact of invasive species, and reveal the factors that underlie the evolution of sociality [1]. Researchers have often examined the ecological correlates of animal social structure, linking variation in social organisation with factors such as habitat type, kin structure, food availability, sexual dimorphism, body size, technological innovation (in human societies) and predation risk [2], [3], [4], [5], [6], [7], [8]. In particular, predation risk is thought to be a key ecological factor driving the evolution of group formation [9]. Nevertheless, despite a long history of studies demonstrating that predation risk is typically associated with larger prey group sizes [8], [10], [11], [12], little is known about how risk shapes the overall social organisation and dynamics of animal populations.

Fission-fusion societies, which describe populations that are characterised by frequent exchange of individuals among groups [13], [14], [15], [16], [17], provide an ideal system in which to examine the ecological correlates of sociality because these systems show rapid responses to changing environmental conditions [18]. While grouping can confer advantages such as increased hunting efficiency, transmission of cultural information and reduced risk of predation, it can also incur potential costs such as increased competition and aggression among group members and greater risk of parasitism [19]. Ecological variability is predicted to drive changes in the relative costs/benefits of group membership, leading to dynamic patterns of fission-fusion [20]. Thus studying the organisational properties of fission-fusion societies not only reveals the factors that influence an individual's grouping decisions, but can also provide insights into how interactions among individuals drive social relationships and ultimately influence population processes [21], [22].

Studies linking the social structure of fission-fusion societies with ecological variability have tended to focus on the distribution, abundance or quality of food resources [17], [23], [24], [25], [26], [27] and its associated effects on competition among individuals within the group [28]. Those studies that have documented a link between predation risk and prey group size have been conducted on populations in the wild [29], [30], [31], where the role of other interacting factors (e.g. food availability) is difficult to determine. For example, divergent social structures observed in communities of Hawaiian dolphins (fission-fusion societies versus stable associations) could partly be attributed to variation in predation risk, but the availability of prey and suitable habitats was also important [32].

Temporal patterns of social organisation, such as the frequency of fission-fusion events, are an important but often overlooked aspect of sociality [33], and these processes form the basis of large scale effects such as rates of gene flow and speciation. A study with bison at risk of predation from wolves found that the probability of fission-fusion events depended on the habitat, season and the time of day at which the bison were observed [34], but studies relating risk to temporal organisation are generally lacking. Despite recent advances in the analytical techniques used to quantify animal social organisation [33], [35], no study has yet adopted an experimental approach for examining the dynamic effects of predation risk on the spatial and temporal organisation of prey populations.

In this paper we use social network analyses to investigate how variation in predation risk affects an individual's grouping decisions and how this contributes to the overall patterns of social organisation. A social network is a graphical representation of the social associations or interactions occurring among individuals within the population(s) [35]. While these analyses originated as a branch of mathematical theory that were applied to fields as diverse as physics [36], systems pharmacology [37], epidemiology [38] and conservation biogeography [39], they have recently attracted the attention of behavioural ecologists [22], [35], [40], [41], [42] because calculation of their mathematical parameters can reveal global properties of the population, such as resilience to the removal of particular individuals [43] and the rate at which disease or learned information might be transmitted [44].

We investigated the effect of predation risk on social organisation by comparing the social structure of populations of freshwater fish (guppies) collected from habitats with high and low predation risk and observed in the lab under standard conditions (no risk). Guppies have proved a tractable system for network studies [14], [45], [46], [47], [48] and provide a compelling example of the effect of predation risk on a variety of phenotypic and behavioural traits [49], [50]. Specifically, classic early work with this species [51] revealed schooling as an evolutionary response to predation, yet we do not know how this group structuring contributes to the overall patterns of social organisation at the population level. For example, different patterns of social organisation may be observed in populations characterised by similar group sizes; between sampling intervals shoal membership may remain stable (low rates of exchange) or be highly dynamic with individuals frequently switching among shoals. These temporal processes are of key importance because they determine how individual movements affect population dynamics [52], such as rates of gene flow, the spread of information or pathogens and the opportunity for social recognition (e.g. familiarity) to develop among individuals.

We anticipated that the larger shoal sizes typically found in high predation guppy populations would produce social networks with higher levels of association among group members than those in low predation habitats. However, we expected temporal patterns of organisation to also play an important part: low rates of fission-fusion are expected to lead to stable, highly associated groups but low connectivity among groups, while high rates will lead to greater movement of individuals among shoals and greater overall connectedness at the level of the community. If movement of individuals (or small groups) among shoals is risky in habitats with high predation risk [53], then within-shoal associations may be high but among-shoal connectedness may be low. In guppies from habitats with low predation risk, within-shoal associations may be low but among-shoal social connectedness may be high if fission/fusion events are frequent. Stable associations can lead to the development of social familiarity among individuals, which can confer foraging and antipredator benefits in fishes [54]. In guppies familiarity takes around 12 days to develop [55]; we therefore used this time frame to determine whether the development of preferred companionships affected the properties of the networks.

## Materials and Methods

### Ethics statement

This work was approved and performed in accordance with guidelines issued by the Animal Care and Ethics Committee (ACEC) for the University of New South Wales, Australia, under research project approval number 05/34A (issued to J.L.K.). Fish were transported from field sites to the laboratory in Trinidad in sealed plastic bags containing river water, plant material collected from the river (to provide cover/refuge) and Aqua Master Armour Coat™ (for skin protection). Fish were acclimatised in the laboratory in their social groups overnight before being individually tagged (see below for details) the following day. These individual marks were necessary to ensure that each female was individually recognisable (wild female guppies do not have distinguishing colour patterns). We ensured that females were anesthetised briefly (in MS222) for the procedure and allowed to recover in aerated and conditioned water (containing Armour Coat™). Fish were allowed to recover for a further 24 hours before experiments commenced, during which the health and behaviour of all fish was carefully monitored. We did not observe any ill effects or changes in behaviour as a result of the tagging procedure. At the end of our experiments, the fish were released into large artificial pools at the research station.

### Fish collection

Guppies in the Northern Range Mountains of Trinidad, West Indies, inhabit a series of streams that can readily be characterised by predation risk because barrier waterfalls have prevented the upstream migration of most predators. Guppies living in the upper reaches of streams occur with only one fish predator, Hart's Killifish (*Rivulus hartii*), which predominantly feeds on juvenile guppies and invertebrates [56], while guppies inhabiting the lower regions of streams coexist with a variety of fish predators including the pike cichlid (*Crenicichla frenata*) the blue acara (*Aequidens pulcher*) the wolf fish (*Hoplias malabaricus*) and Hart's killifish (*Rivulus hartii*) [57].

We collected guppies from twelve localities in Trinidad comprising habitats with high (n = 7) and low (n = 5) predation risk. Ten of these sites were upstream (low predation risk) and downstream (high predation risk) populations for the same river (Arima, Aripo, Guanapo, Tacarigua and Turure Rivers). We also collected guppies from a river (the Oropuche) in which upstream and downstream sites are both known to be high predation. Inclusion of these populations allowed us to consider whether any differences observed between upstream/downstream populations might be attributed to ecological parameters other than predation risk. Predator assemblages at all our sample sites have been determined previously and fit the typical upstream (low risk) and downstream (high risk) pattern (see Table S1 for details of collection sites and the predator assemblages).

A seine net was used to collect 8–10 shoals of adult guppies per population along approximately 50 m stretch of each river. The shoals were placed in a single bucket so that fish from each population were derived from mixed shoals and were unlikely to be familiar with one another. We then removed 12 similar-sized females before returning all remaining fish to the river. As the size distribution of females covaries with predation risk [58], we collected females that had a body size that lay between the means for high and low predation risk environments (mean total length of fish used in this study  = 26.9±0.30 mm). Females were returned to the laboratory at William Beebe Tropical Research Station (Simla) in Trinidad, where each population was released into a separate circular observation arena (diameter  = 120 cm, water depth  = 5 cm) containing rainwater and left to acclimate overnight.

### Quantifying social associations

We constructed a total of twelve social networks, each comprising 12 females collected from the same population (seven high predation and five low predation sites, n = 144 individuals). We chose to use only females in this study to avoid confounding general association (shoaling) behaviour with sexual behaviour. Furthermore, previous work on guppy social networks has revealed persistent pairwise associations and cooperative interactions predominantly among females [14], [45]. Groups of 12 fish were chosen for each population because of the difficulty of observing the behaviour of a large number of individuals. In addition, laboratory-based network studies utilising small numbers of individuals are a good predictor of association patterns occurring in larger networks constructed in the wild [45]. Small guppy shoals are common in the wild [59] and a sample size of 12 individuals allows for evaluation of associations among several social groups.

As the construction of social networks requires that each individual is recognisable, we anesthetised each female in MS222 and gave her a unique identification tag by injecting VIE (Visible Implant Elastomer, Northwest Marine Technology, Inc.) dye under the surface of the dorsal epidermis [59]. The total length (TL) of each fish was then measured (to the nearest millimetre) using a pair of callipers and all fish were left to acclimate overnight in their respective pools. The following day we observed associations among individuals once per minute over a 30-minute period; previous work has shown that this period is sufficient for revealing non-random social structure in guppies [14]. Females were considered to be associating if they were found within the same shoal, i.e. within four body lengths of one another, a standard method of evaluating shoaling behaviour [60]. Individuals were considered part of the same shoal provided they were within 4 body lengths of another group member (thus a shoal of 3 fish could be 8 body lengths wide). Wild guppy shoals are highly dynamic with shoals exhibiting fission or fusion events (i.e. at least one individual joining or leaving a shoal) on average every 14 s in a population with high predation risk [59]. We chose a considerably longer sampling interval (1 minute) so that sampling periods for high and low predation populations were likely to be independent, as required by social association models [61]. Observations were repeated every 3 days over a 12-day period ( =  total of 4 observations per population), the time scale over which familiarity has been shown to develop in the laboratory in this species [55]. A total of 41 social networks were therefore constructed and modelled (7 networks were not sampled at the allotted time due to time constraints).

### Testing for non-random patterns of social organisation

The social analysis program Socprog 2.3 [33] was used to calculate several measures of association describing different aspects of social structure: association index (AI), gregariousness and population social differentiation (see Suporting Information S1, for further details). AIs provide an estimate of the proportion of time two individuals spend together (0 = not associated, 1 = always associated) [22]. We also calculated gregariousness, which is the mean size of group that an individual experiences [6]. Social differentiation was used as a measure of the variation in associations in our populations: low values (<0.3) suggest little variation and represent homogenous societies while higher values indicate communities that are well differentiated (>0.5) or extremely differentiated (>2.0) [33]. Power analyses were performed to provide the level of confidence at which the true social system has been detected.

We examined whether patterns of social association observed in each of our guppy populations were significantly different from random using the permutation tests in Socprog 2.3. The randomisations were performed for each population because these methods rearrange the empirical data (e.g. row and column sums in the matrices remain the same) so that the overall structure of the data is conserved (in this case, the number of groups each fish is observed in – which may vary among populations). The methods used by Socprog 2.3 have been modified to account for problems that were identified with earlier randomisation techniques [33], [62] Non-random social organisation is expected when the coefficient of variation (CV) of association indices is significantly higher or lower in the real data set than in the random data. Permutation tests were also used to examine population differences in gregariousness with the null hypothesis being that all individuals prefer groups of similar size (standard deviations of typical group size for real and random data are similar). These permutations can also be used by Socprog to identify pairs of individuals whose association index is greater than 97.5% or less than 2.5% of their random association indices indicating dyads with significant preferences and avoidances respectively. We performed 5000 permutations for each population (at which point P-values stabilised to within 0.01) with 1000 trials per permutation.

### Network analyses

We used five network measures which are particularly useful for the analysis of networks based on association indices [22]: strength (or degree), eigenvector centrality, reach, the clustering coefficient and affinity. Such measures have previously been used to describe social organisation in small experimental populations of animals [63]. The strength of the network (referred to as ‘degree’ in binary networks) is the sum of association indices with all other individuals minus one and is equivalent to gregariousness [64]. Eigenvector centrality [65] is a measure of how well an individual is associated with others (in terms of the number and strength of connections), and also how well its neighbours are themselves associated. Eigenvector centrality therefore provides a measure of connectedness of individuals within a network. Reach describes how well an individual is indirectly connected to others in the population and reveals the overall strength of an individual's neighbours. The clustering coefficient describes how well an individual's neighbours are connected to one another. Values of zero suggest that none of an individual's neighbours are associated whereas a value of one indicates that all neighbours are linked [22]. Affinity is the weighted mean strength of an individual's neighbours and is weighted by the association index between them. Mean network measures were calculated for each population in Socprog 2.3 and their associated standard errors were calculated using the bootstrap method with 1000 replicates.

### MANOVA Models

Prior to testing for an effect of predation risk and time (day) on the association measures and network statistics, we used Principal Component Analysis to look for correlations in our data and reduce the number of variables in our model. We entered population means (averaged over the 12 individuals per population) for association index, gregariousness, social differentiation, strength, eigenvector centrality, reach, clustering coefficient and affinity as factors. Two independent principal components (PC1 & PC2) were extracted from the remaining variables (eigenvalues>1.0), which explained 68.5% and 20.9% of the variance in our data respectively. The first Principal Component (PC1) was loaded by the means of association index, gregariousness, strength, eigenvector centrality, reach, clustering coefficient and affinity (loadings: 0.51–0.99). The second principal component (PC2) was predominantly loaded by mean social differentiation (loading: 0.90); we therefore used the raw data for this response variable.

We used MANOVA with the dependent variable as a repeated measures response (4-levels: days) to examine between-subject (predation risk) and within-subject (day) effects on PC1 and mean population social differentiation. The dependent variables were checked for assumptions of normality, homogeneity of variance and sphericity prior to conducting the statistical tests, which were performed in JMP version 9. We confirmed that mean body size of individuals within each group (population) had no effect on our dependent variables by entering it as a covariate in a MANCOVA (PC1: F_1, 6_ = 0.410, P = 0.546; mean population social differentiation: F_1, 6_ = 1.103, P = 0.334). This term was therefore excluded from subsequent statistical tests.

### Temporal patterns of association

Temporal patterns of association were examined using lagged association rates, which describe the probability that two individuals will associate τ time units after a previous association [33], [66]. Lagged association rates were plotted continuously (using moving averages) against time lag in Socprog 2.3. We considered changes in lagged and null association rates over two different time scales: time lag in minutes during the 30-minute observation period and time lag in days over the 13-day study period. The precision of lagged association rates was estimated by jacknifing across sampling periods, using a 5-minute sampling period for ‘observation’ (7 jackknife groups) and a 1-day sampling period for ‘day’ (4 jacknife groups).

The lagged association graphs were fitted to several mathematical models in order to describe the temporal pattern of organisation in our populations and estimate the parameters of the models. To reduce the number of mathematical models fitted to the data we first inspected the lagged association rate curves to determine the descriptors that were most appropriate. For sampling periods in minutes, we chose four exponential models (Table 1) that included rapid periods of disassociation (breaking up of groups), as these would be expected in fission-fusion societies [22]. We selected the model of best fit for each population according to the quasilikelihood variant of the Akaike Information Criterion (QAIC) where the QAIC value provides an indicator of the level of support for the model (lower values providing the highest level of support) [67]. For time periods of days, inspection of the lagged association rates revealed linear relationships; we therefore fitted a custom model 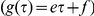 describing a constant rate of change in association probability over time (Table 1).

**Table 1:** Description of models fitted to lagged association rates (g) in Socprog 2.3. For time lags (τ) of minutes, we chose four models (parameters: *a, b, c, d*) that incorporated rapid periods of disassociation and selected the model of best fit according to the quasiliklihood Akaike Information criterion (QAIC).

Name		Model	Description
RD + CC	Rapid disassociation + constant companions	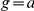	Some associations decay within 1 sampling period then g is stable. Short-lived, non-random associations
RD + CA	Rapid disassociation + casual acquaintances	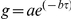	Some associations decay within one sampling period then g falls to zero.
RD+CC+CA	Rapid disassociation + constant companions + casual acquaintances	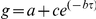	Rapid disassociation within one sampling period and an association rate that falls before levelling off.
RD+2CA	Rapid disassociation + 2 levels of casual acquaintances	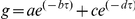	Rapid disassociation within one sampling period and levels of disassociation at time intervals of 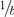 and 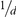 .
Custom	Gradual change in association/avoidance over time	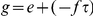	Linear change in probability that two individuals remain associated following time lag.

For time lags of days we fitted a custom model describing a linear change in association probability over time.

To compare temporal patterns of social organisation among high and low predation risk populations, the overall best-fitting model was applied to all populations and the final model's parameters calculated. We then compared the parameters from these best-fit models (using t-tests) to evaluate the effect of predation risk on patterns of temporal organisation. In order to compare the temporal structure of our populations we had to find the overall model of best-fit; our aim of this part of the study was therefore not to construct accurate mathematical models, but to look for differences in overall patterns of temporal organisation in populations from high and low risk environments.

## Results

### Testing for non-random patterns of social organisation

Females from both high and low predation risk populations exhibited non-random patterns of social organisation with some individuals preferentially associating with or avoiding others (coefficients of variation for the real association indices were significantly different from those for the random indices; high risk real CV = 0.52, random CV = 0.37, P = 0.01; low risk real CV = 0.77, random CV = 0.56, P = 0.01). Inspection of the dyadic levels of significance revealed that overall, paired associations were characterised by patterns of avoidance (indicated by P<0.025) rather than preference (P>0.975). Patterns of gregariousness did not differ from those expected from random models (i.e. there was no preference by individuals for large or small group sizes); standard deviations of typical group size for real and random data sets were similar for both high (sd real data = 0.70, sd random = 0.52, P = 0.70) and low predation populations (sd real data = 0.77, sd random = 0.55, P = 0.85).

### Effect of predation risk on social networks and their development

**Figure 1:**
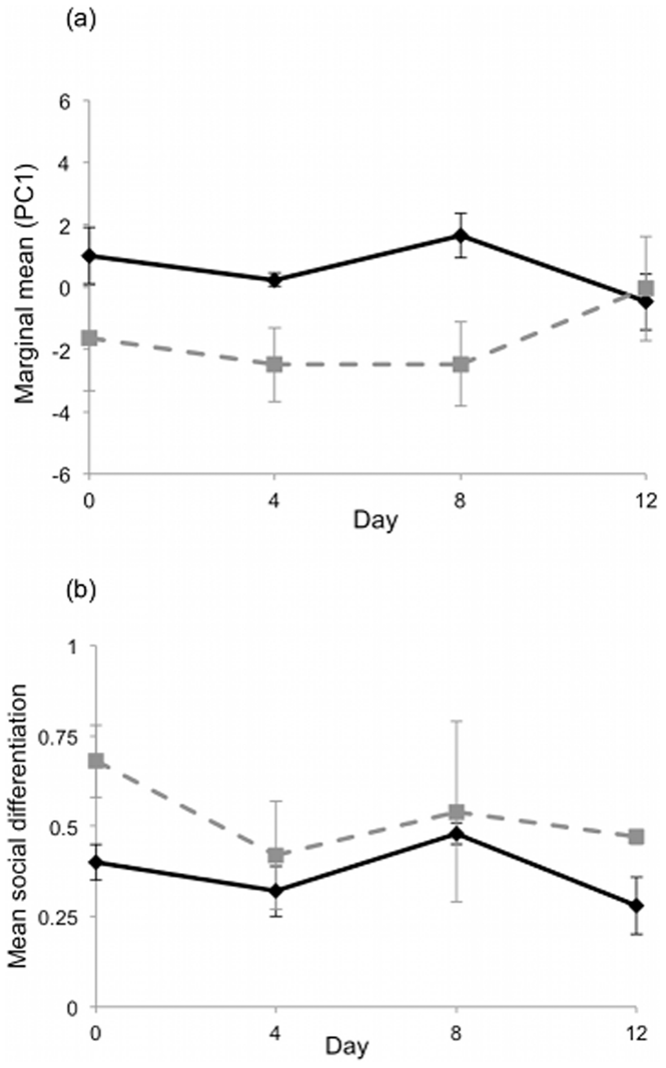
Effect of time (day) and predation risk on guppy social network measures. Figure a represents the marginal means (± SE) for PC1 while figure b shows the mean population social differentiation. Solid lines represent high predation populations; dashed lines are low risk sites.

**Figure 2:**
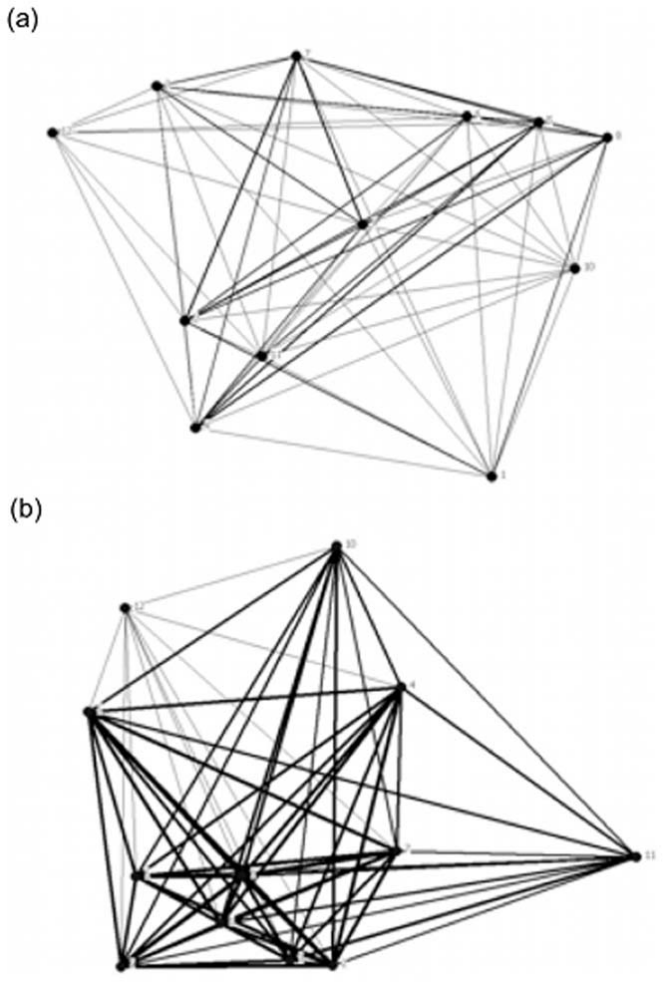
Example of social networks for female guppies collected from habitats differing in predation risk. Fish were from a low predation (a) and high predation (b) population of the Tacarigua River, Trinidad. Associations are represented by lines (edges) between individuals, which are weighted so that stronger associations are shown with darker lines. Drawn in Netdraw [91].

We found a significant effect of predation risk on PC1 and mean population social differentiation. The variables loaded by PC1 – population means for association index, gregariousness, strength, eigenvector centrality, reach, clustering coefficient and affinity - had higher mean values in high predation populations than low predation ones (Fig 1a, Tables 2 & 3). Overall our populations showed some level of social differentiation (values >0.3) and this effect was stronger for low risk populations than high risk ones (Tables 2 & 3; Fig. 1b). There was no effect of day or the (predation risk x day) interaction term on PC1 or mean population social differentiation in our social association networks (Table 2). Thus overall, the social networks for females from low predation risk environments (Fig. 2a) had fewer connections with lower overall strength than those for fish from high predation risk habitats (Fig. 2b). We accounted for sampling upstream and downstream locations of the same stream by entering both river (6-levels) and day as random effects in a General Linear Mixed Model with predation risk as the fixed factor (it was not possible to have random effects in the MANOVAs). The random effect was non-significant for both PC1 and social differentiation (F_5, 31_ = 2.04, P = 0.10 and F_5, 31_ = 0.86, P = 0.52 respectively); the effect of predation risk in this model remained significant for PC1 (F_1, 31_ = 8.50, P = 0.007) but not social differentiation (F_1, 31_ = 0.25, P = 0.62).

**Table 2:** MANOVA models testing the effects of predation risk, day and their interaction on PC1 and mean population social differentiation (both entered as repeated measures responses).

*Response variable*	*Dependent variable*	*df*	*F*	*P*
PC1	Predation risk	1, 7	5.77	**0.047**
	Day	3, 21	0.26	0.857
	Predation risk *day	3, 21	1.41	0.269
Social differentiation	Predation risk	1, 7	11.39	**0.012**
	Day	3, 21	1.15	0.353
	Predation risk*day	3, 21	0.37	0.779

Significant effects at P<0.05 are shown in bold.

**Table 3:** Network association measures averaged over sampling periods (days) and populations to give overall means and standard errors for high (n = 7) and low risk (n = 5) networks.

Measure	*High predation*	*Low predation*
Association index (AI)	0.265±0.03	0.177±0.03
Gregariousness	0.847±0.08	0.680±0.07
Social differentiation	0.395±0.03	0.414±0.06
Strength	2.911±0.27	1.95±0.29
Eigenvector centrality	0.276±0.001	0.271±0.003
Reach	10.95±1.82	5.69±1.54
Clustering coefficient	0.53±0.03	0.45±0.03
Affinity	3.16±0.29	2.14 ±0.29

Data were obtained from Socprog 2.3.

### Temporal patterns of association

**Figure 3:**
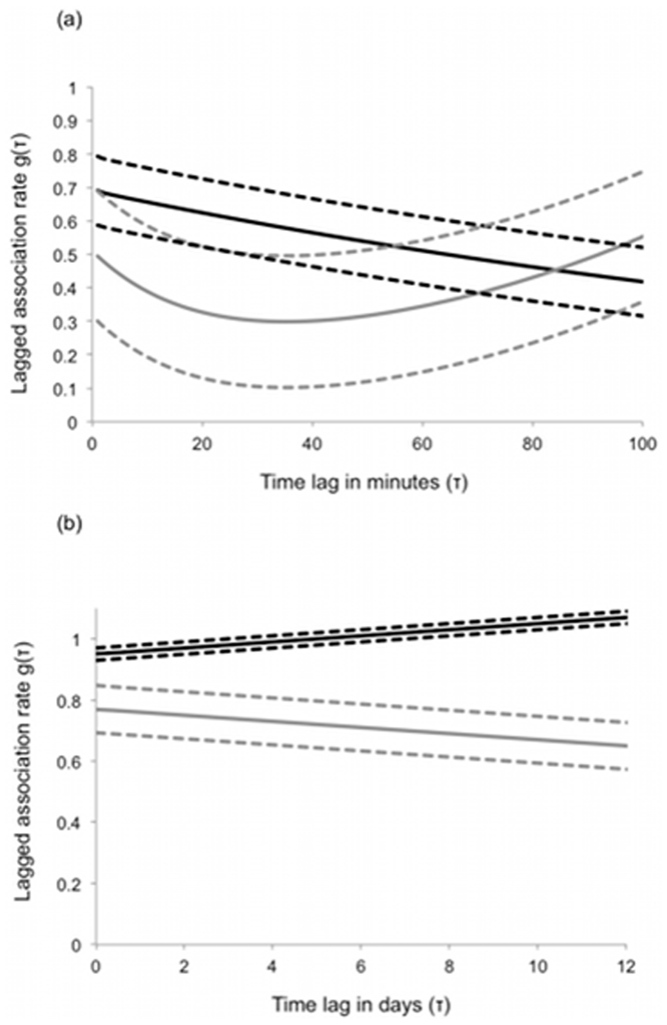
Models of temporal social structure in guppy populations. Lagged association rates are plotted against time lag in minutes (a) and days (b) for networks from high (black lines) and low (grey lines) predation populations. Solid lines indicate mean parameter values; dotted lines indicate the upper and lower boundaries for the standard error of the mean parameter values. The x-axis extends beyond the 30-minute sampling period (in fig. a) to illustrate the longer, 2nd period of fission predicted by the models.

Lagged association rates plotted for both high and low predation risk populations were best described by an exponential model of rapid disassociation with two levels of casual acquaintances (RD+2CA); QAIC values were lowest for this model in 7 of our 12 populations (the RD + CA model best-fit all the remaining populations except one). In this model 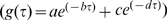
parameters *a* and *c* are the proportion of associations that break up within a sampling period and 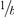 and 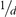 represent the time scale of these two periods of disassociation. Thus the first term in the model 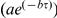
represents fission events over short time scales while the latter term 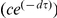 describes longer term changes in social organisation [22]. This type of model may represent fission of short term associations and the slow decay of more permanent relationships or might arise where some individuals form stable groups that move in and out of other groups [22]. The observed association rates were higher than the null rates in each of our populations, confirming that females displayed preferences/avoidances for particular individuals over both time scales (the 30-minute observation period and the 13-day duration of the study). Fitting the RD +2CA model 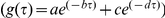
to each of our populations and calculating mean values of the model's parameters gave the following estimates of lagged association rates for high and low risk populations (Fig. 3a):
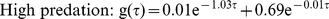


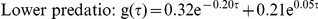



Comparison of the estimated parameters of our lagged association models revealed that predation risk contributed to varying patterns of temporal social organisation. For the first term in the model 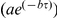
representing changes in short term associations, parameters *a* and *b* differed significantly between populations with high and low predation risk. At short time lags, associations between pairs of low predation females were more likely to break up than those between high predation fish (parameter *a*, mean ± s.e.: high risk = 0.01±0.05, low risk = 0.32±0.10; t_10_ = 2.23, P = 0.014). The time scale over which these short-term fission events occurred (given by 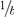) was also influenced by predation risk; in other words, associations among fish from high predation populations tended to break up over periods of less than one minute while those among fish in low predation populations broke up over periods of around 5 minutes (parameter *b*, mean ± s.e.: high risk = 1.03±0.09, low risk = 0.20±0.15, t_10_ = 2.23, P<0.001).

For the second period of disassociation 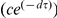 the above patterns were reversed. Affiliations between pairs of high predation females were more likely to break up than those between low predation fish (parameter *c*, mean ± s.e.: high risk = 0.69±0.05, low risk = 0.21±0.10, t_11_ = 2.20, P = 0.001). Indeed, the model for fish from low predation habitats suggests an increase in the probability of association over longer time periods (e.g. >40 mins; Fig 3a). Here, the models suggest that associations among fish from high predation habitats broke up over periods of 100 minutes while fish from low predation habitats disassociated over a period of about 20 minutes. However, the error around these estimates was high and the differences not significant (parameter *d*, mean ± s.e.: high risk = 0.01±0.00, low risk = -0.05±0.04, t_11_ = 2.20, P = 0.10). The effect of predation risk on parameters *a, b, c* remained significant following correction for multiple comparisons [False Discovery Rate for dependent comparisons, 68; adjusted critical significance value  = 0.024]. When we considered patterns of association over the 13-day familiarity period, predation risk did not influence rates of change in association with time (Fig. 3b; parameter *e*, high risk  = 0.01±0.00, low risk  = -0.01±0.01, t_8_ = 2.31, P = 0.19).

## Discussion

Our results reveal that predation risk can influence the fine-scale social structure of fission-fusion societies. While a long history of studies has examined the relationship between predation risk and prey group size [5], [6], [69], [70], our findings suggest that more complex elements of social dynamics can be influenced by risk. Specifically, we found that individuals from high predation risk populations were more closely affiliated, and also their neighbours better socially connected, than those from low risk populations that had weaker social ties. Our results also provide one of the first demonstrations of the link between predation risk and temporal patterns of social organisation. Associations among fish from low predation habitats broke up over periods of 5 and 20 minutes, while fish from high predation habitats were more likely to remain associated during these time frames. Thus the strong associations recorded among fish from high predation rivers during our 30-minute observation period likely contributed to the higher levels of overall social connectedness in high predation networks versus low predation ones. Interestingly, the lagged association models suggested that the reverse pattern occurred over longer time periods with associations among fish from low predation rivers more likely to persist. We therefore suggest that the stability of affiliations among females crucially depends on the time frame over which these social preferences are observed.

The dynamic nature of fission-fusion societies provides an ideal framework for testing socioecological theory - the identification of ecological factors that drive variation in social behaviour - because these can provide key insights into large scale evolutionary processes [70], [71]. Previous applications of social analysis techniques to wild animal populations have revealed the importance of ecological factors in influencing social organisation; for example, seasonal changes in resource availability can influence the social structure of baboon, bat and elephant societies [17], [24], [72]. While several studies have considered the role of predation risk in contributing to the observed community social structure [32], [34], [73], our study provides the first experimental approach to examining the effects of evolutionary/ontogenetic exposure to predation risk on animal sociality.

We predicted that due to the high risk of predation associated with the movement of individuals among groups, shoals collected from high predation environments would exhibit higher stability with reduced frequency of fission-fusion events compared with fish in low predation habitats. Thus we expected that high levels of association would be observed within shoals but lower levels of overall social connectivity would occur among these groups. Our findings do not appear to match these predictions because we found greater overall social connectivity in fish from high predation risk habitats (in our 30-minute observation period). One explanation for this is that our sample populations represented associations among a small number of shoals (n = 12 individuals; median natural shoal size = 5 fish) in a contained area, thus individuals are likely to be more associated than fish in the wild that have greater opportunity for movement among different social groups. Another possible explanation that requires further investigation is that fission/fusion events may involve movements of different sized groups. In fish collected from high predation habitats, small groups of fish may move among larger shoals to reduce the risks associated with travelling among groups. This would likely contribute to high overall levels of social connectivity at the community level. In fish from less risky habitats, individuals may be more likely to move in and out of groups. More research into the temporal dynamics of shoaling behaviour would shed light into mechanisms of movement within and among shoals.

We acknowledge that a limitation of our study (and a common problem in many other social network studies) is the lack of within-population replication. In order to generalise our findings and confidently describe the social structure of a particular population, we would need to construct multiple, independent networks for each sample site. However, this was not the purpose of our study (our networks were constructed in small artificial pools in the laboratory); rather, we aimed to look for overall effects of risk on social behaviour at the level of the population. Whilst we are confident that the divergent patterns of social structure observed in this study correspond to variation in predation risk, we suggest that within-population variation in social organisation is an important area for future research.

It is possible that factors other than predation risk varied between upstream and downstream sites (e.g. population density, sex ratio, water quality, canopy cover) and contributed to our observed differences in social structure. However, we attempted to control for any of these immediate effects by observing same-sex networks comprising similar-sized individuals in the laboratory under identical conditions. Inspection of the association indices for females from upstream and downstream populations of the Oropuche River, which are both high predation environments [74], reveals that they are similar (e.g. mean AI ± se: upstream = 0.18±0.04, downstream  = 0.19±0.03), giving us some confidence that differences among populations are a result of predation risk, rather than any other ecological variables. Predation risk is known to influence not only group size and shoaling tendency in guppies, but also a number of other social behaviours such as cooperation, microhabitat use and courtship [50]. Thus the relationship between social organisation and predation risk reflects the combination of the direct and indirect social implications of risk. Like previous work describing the effects of predation risk on guppy shoaling behaviour [11], our observations were also conducted in the absence of predator-related cues, suggesting a fundamental basis to the observed variation in sociality. While our findings are a necessary first step in evaluating the effects of prior predation risk on spatial and temporal social structure, it would be very interesting to investigate the effects of immediate risk (e.g. predator presence) on social structure in fish populations from high and low risk habitats [75].

Relating the structure of social networks to their large-scale properties [76] allows us to make a number of predictions regarding the effect of predation risk on large-scale population processes. Studies linking social organisation with disease transmission have revealed that individuals that play a central role in the network are more likely to become naturally infected with pathogens than those that are less associated [77]. Furthermore, experimental infection of highly socially connected individuals can cause higher rates of disease transmission than if randomly selected individuals are infected [78]. Translating these findings to the present study, we might predict that the transfer of disease or information would be more rapid in high predation populations where individuals are better connected and likely to remain associated, at least over short time periods. Guppies can learn novel foraging tasks, escape routes and antipredator responses through associating with or following other individuals [79], [80] and learn more effectively from familiar fish than unfamiliar fish [81]. Specifically, guppies behave differently in the presence of trained/experienced fish and the novel behaviour is retained once they (the trained fish) are removed. Thus, novel information is expected to spread through a population depending on the movements of the knowledgeable/experienced individual(s) and their social connections within the community [82].

The flow of information/disease is likely to be non-random and directed through particular individuals or sub-groups within the community [83]. Previous studies have shown that age [84], [85], kinship [73] and behavioural phenotype [48], [86] can bias individual-level interactions and affect the centrality of the network [87]. Identifying the role that these genetic and phenotypic factors play in structuring both high and low predation risk networks is an interesting avenue for further research. The level of sub-structuring or ‘cliquishness’ within the community is important because information or disease may be transmitted rapidly through highly connected sub-groups but less rapidly through the population as a whole [76]. The level of sub-structuring observed in this study (given by the clustering coefficient; population range: 0.4 to 0.74) is higher than that reported for dolphins and sperm whales [88], [89] and similar to that reported for bats and other freshwater fishes [47], [90]; high levels may act to facilitate the flow of information among members of cliques while increasing their susceptibility to disease. Cliquish networks are also predicted to be less robust as the removal of key individuals or their associations may cause the community sub-structure to fragment [42].

We found no evidence that increasing familiarity among females, which occurs over the course of 12 days in guppies [55], influenced the development of the social networks. This is in contrast to previous work showing that familiarity can develop in small networks comprised of randomly selected females [46]. In this previous study [46], the development of social recognition was examined by testing the preference of females for shoal partners originating from the same social network over females from different networks. One suggestion for the discrepancy in results between our study and that of Darden et al. [46] is that the effects of increasing familiarity among females are not evidenced by changes in association towards one another but rather by their response upon encountering unfamiliar individuals. Thus social recognition among small groups of individuals may develop in the absence of any observed changes in overall network structure over time.

Understanding the impacts of ecological variability on animal social organisation is essential because it allows us to identify factors that may have facilitated the evolution of sociality. The recent advancement of social analysis techniques allows us to go beyond describing the effects of predation risk on prey group size and composition to reveal increasingly complex patterns of spatial and temporal organisation. Our findings suggest that the social structure of a population depends on the temporal scale at which patterns of association are observed. Consideration of the temporal aspects of social organisation is therefore essential, particularly in the case of fission-fusion societies where group splitting/joining events can occur on multiple time scales.

## Supporting Information

Table S1 Details of the predator assemblages at each of the twelve guppy populations sampled.
(DOC)

Supporting Information S1 Additional information on the analysis of social structure using Socprog.
(DOC)
