# Supplementary material for: Predation Risk Shapes Social Networks in Fission-Fusion Populations
Source: PLoS One. 2011 Aug 30;6(8):e24280. doi: 10.1371/journal.pone.0024280 (PMC3166168; doi:10.1371/journal.pone.0024280)
Supplement: Table S1 — Details of the predator assemblages at each of the twelve guppy populations sampled. (DOC) [file pone.0024280.s001.doc]

**Table S1**

| Population | Grid reference | Predrisk | Predator assemblage |
| --- | --- | --- | --- |
| Upper Aripo | 10 ^o^ 41.74’ N 61 ^o^12.41’ W | Low | R ^2^ |
| Lower Aripo | 10 ^o^ 39.04’ N 61 ^o^13.38’ W | High | C ^2^ low densities |
| Upper Arima | 10^o^ 43.05’ N 61 ^o^ 17.89’ W | Low | R ^1^ |
| Lower Arima | 10 ^o^ 39.03’ N 61 ^o^ 17.39’ W | High | C, A ^4^ |
| Upper Guanapo | 10 ^o^ 41.60’ N 61 ^o^ 15.80’ W | Low | R ^3^ |
| Lower Guanapo | 10 ^o^ 39.50’ N 61 ^o^15.20’ W | High | C, A, Ho, As, He ^1,3^ |
| Upper Turure | 10 ^o^ 40.78’ N 61 ^o^10.00’ W | Low | R ^3^ |
| Lower Turure | 10 ^o^ 39.39’ N 61 ^o^10.09’ W | High | C, A, Ho, As, Ci ^3, 4^ |
| Tunapuna | 10^o^ 40.11’ N 61 ^o^ 23.39’ W | Low | R ^2^ |
| Middle Tacarigua | 10^o^ 41.39’ N 61 ^o^ 21.52’ W | High | C, A, Ho, As, He ^2, 4^ |
| Upper Oropuche | 10 ^o^ 43.06’ N 61 ^o^ 08.80’ W | High | C, A, Ho, As, Ci ^1^ |
| Lower Oropuche | 10 ^o^ 39.58’ N 61 ^o^ 07.88’ W | High | C, A, Ho, As, Ci ^1^ |

Predator codes: R=*Rivulus hartii,* C=*Crenicichla frenata,* A=*Aequidens pulcher,* Ho=*Hoplias malabaricus,* As=*Astyanax bimaculatus,* He=*Hemibrycon taeniurus,* Ci=*Cichlasoma taenia.*

**References**

1. Endler JA, Houde AE (1995) Geographic variation in female preferences for male traits in *Poecilia reticulata*. Evolution 49: 456-468.

2. Magurran AE, Seghers BH (1994) Sexual conflict as a consequence of ecology: evidence from guppy, *Poecilia reticulata,* populations in Trinidad. Proceedings of the Royal Society London B 255: 31-36.

3. Magurran AE, Seghers BH (1991) Variation in schooling and aggression amongst guppy (*Poecilia reticulata*) populations in Trinidad. Behaviour 118: 214-234.

4. Botham MS, Hayward RK, Morrell LJ, Croft DP, Ward JR, et al. (2008) Risk-sensitive antipredator behavior in the Trinidadian guppy, *Poecilia reticulata.* Ecology 89: 3174-3185.
