# Supplementary material for: Predation Risk Shapes Social Networks in Fission-Fusion Populations
Source: PLoS One. 2011 Aug 30;6(8):e24280. doi: 10.1371/journal.pone.0024280 (PMC3166168; doi:10.1371/journal.pone.0024280)
Supplement: Supporting Information S1 — Additional information on the analysis of social structure using Socprog. (DOC) [file pone.0024280.s002.doc]

**S2**

*S2.1. Calculation of association indices and population social differentiation*

We used the *simple ratio* index of association which is the number of sampling periods in which a pair is observed together divided by the number of samples in which either individual was observed . This index is particularly suited to studies in which all individuals are identified at every sampling period (no immigration/emigration) and associations are symmetric (i.e. the association index of fish A and B is equal to that of fish B and A) . Fish were considered to be associated if they occurred in the same shoal during the sampling period (AI = 1) and not associated (AI = 0) if they were not in the same shoal for that sampling period. Each observation formed a sampling period so that there were 30 sampling periods for each population per day.

Social differentiation was calculated by removing an estimate of the sampling variance from the coefficient of variation of the estimated association indices . Highly differentiated societies may have some weak associations and a few very strong relationships. The power of this analysis was calculated by estimating the correlation coefficient between the true association indices (the actual proportion of time pairs spend associating) and the calculated association indices (estimates of these association times) using the likelihood method. Power values range between 0 and 1 with high values suggesting high confidence that the true social system has been detected by the analysis.

*S2.2. Testing for non-random associations*

We permuted a group-by-individual matrix to test the null hypothesis that there are no preferred or avoided companionships given the total number of groups each individual was observed in during the (30-minute) observation period . We calculated mean AIs and coefficients of variation for both real and random (permuted) data sets and examined the P values for each. Populations with persistent pairwise associations were characterized by a significantly high or low coefficient of variation in the real data sets compared with the random ones at P<0.05 [6]. Dyads whose association was greater than 97.5% or less than 2.5% of their random AIs, were considered to exhibit significant preference and avoidance respectively. We identified the number of preferred/avoided companionships for populations where the CV was larger in the real data set than the random ones.

Variation in gregariousness among individuals from each of our populations was calculated using the standard deviation of typical group size for both real and random (permuted) data and the corresponding P-values [7]. P-values greater than 0.95 suggest that the standard deviation of the real data is greater than that for the random data, i.e. some individuals in the population are found in consistently larger or smaller groups than other individuals. In these cases, we identified the number of individuals found in significantly larger (where P>0.975) or smaller (P<0.025) groups than expected.

**References**

1. Ginsberg JR, Young TP (1992) Measuring association between individuals or groups in behavioural studies. Animal Behaviour 44: 377-379.

2. Cairns SJ, Schwager SJ (1987) A comparison of association indices. Animal Behaviour 35: 1454-1469.

3. Whitehead H (2008) Analyzing animal societies. Chicago: University of Chicago Press. 336 p.

4. Bejder L, Fletcher D, Brager S (1998) A method for testing associative patterns of social animals. Animal Behaviour 56: 719-725.

5. Whitehead H, Dufault S (1999) Techniques for analysing vertebrate social structure. Advances in the Study of Behavior 28: 33-74.

6. Whitehead H (2009) SOCPROG programs: analysing animal social structures. Behavioural Ecology and Sociobiology 63: 765-778.

7. Whitehead H, Bejder L, Ottensmeyer AC (2005) Testing association patterns: issues arising and extensions. Animal Behaviour 69: e1-e6.
